# How Accurate Is Your Activity Tracker? A Comparative Study of Step Counts in Low-Intensity Physical Activities

**DOI:** 10.2196/mhealth.6321

**Published:** 2017-08-11

**Authors:** Parastoo Alinia, Chris Cain, Ramin Fallahzadeh, Armin Shahrokni, Diane Cook, Hassan Ghasemzadeh

**Affiliations:** ^1^ School of Electrical Engineering and Computer Science Washington State University Pullman, WA United States; ^2^ Geriatrics / Gastrointestinal Oncology Service Memorial Sloan-Kettering Cancer Center New York, NY United States

**Keywords:** activities of daily living, activity tracker, mobility limitations, mobile health

## Abstract

**Background:**

As commercially available activity trackers are being utilized in clinical trials, the research community remains uncertain about reliability of the trackers, particularly in studies that involve walking aids and low-intensity activities. While these trackers have been tested for reliability during walking and running activities, there has been limited research on validating them during low-intensity activities and walking with assistive tools.

**Objective:**

The aim of this study was to (1) determine the accuracy of 3 Fitbit devices (ie, Zip, One, and Flex) at different wearing positions (ie, pants pocket, chest, and wrist) during walking at 3 different speeds, 2.5, 5, and 8 km/h, performed by healthy adults on a treadmill; (2) determine the accuracy of the mentioned trackers worn at different sites during activities of daily living; and (3) examine whether intensity of physical activity (PA) impacts the choice of optimal wearing site of the tracker.

**Methods:**

We recruited 15 healthy young adults to perform 6 PAs while wearing 3 Fitbit devices (ie, Zip, One, and Flex) on their chest, pants pocket, and wrist. The activities include walking at 2.5, 5, and 8 km/h, pushing a shopping cart, walking with aid of a walker, and eating while sitting. We compared the number of steps counted by each tracker with gold standard numbers. We performed multiple statistical analyses to compute descriptive statistics (ie, ANOVA test), intraclass correlation coefficient (ICC), mean absolute error rate, and correlation by comparing the tracker-recorded data with that of the gold standard.

**Results:**

All the 3 trackers demonstrated good-to-excellent (ICC>0.75) correlation with the gold standard step counts during treadmill experiments. The correlation was poor (ICC<0.60), and the error rate was significantly higher in walker experiment compared to other activities. There was no significant difference between the trackers and the gold standard in the shopping cart experiment. The wrist worn tracker, Flex, counted several steps when eating (*P*<.01). The chest tracker was identified as the most promising site to capture steps in more intense activities, while the wrist was the optimal wearing site in less intense activities.

**Conclusions:**

This feasibility study focused on 6 PAs and demonstrated that Fitbit trackers were most accurate when walking on a treadmill and least accurate during walking with a walking aid and for low-intensity activities. This may suggest excluding participants with assistive devices from studies that focus on PA interventions using commercially available trackers. This study also indicates that the wearing site of the tracker is an important factor impacting the accuracy performance. A larger scale study with a more diverse population, various activity tracker vendors, and a larger activity set are warranted to generalize our results.

## Introduction

Increasing physical activity (PA) level and maintaining a healthy diet are among the important factors to sustain and improve cardiovascular health [1-3]. Measuring daily PA is important to objectively monitor an individual’s health. A simple, yet effective, approach to measure PA is to count the number of steps an individual takes in a given day [4]. Based on a common PA guideline, healthy adults are recommended to take 10,000 steps per day to maintain physical fitness and health [5]. However, common recommended levels of PA are challenging targets for less active individuals [6]. Various accelerometer-based devices such as Jawbone, Fitbit, Misfit, and Garmin have been developed for PA monitoring. These devices are small, non-invasive, easy-to-use, and provide an objective indicator of PA behavior [7]. Furthermore, they intend to avoid common sources of error in subjective measurement (eg, self-reporting) [4,8-10]. As researchers continue to utilize the commercially available PA trackers in clinical trials, there is a need to assess the accuracy of these trackers, particularly for low-intensity PAs and those that involve assistive devices [11-13]. Currently, the research community remains uncertain about how reliable off-the-shelf trackers are while performing the aforementioned PAs [14].

There have been several studies analyzing these trackers of which some are aimed at older adults and patients with chronic diseases [4,5,7-10,12,15-25]. These studies evaluated validity and reliability of activity trackers such as Fitbit (ie, Zip, One, Ultra, and Flex) during slow, moderate, and brisk walking in laboratory settings or free living environments. These research studies mostly utilized Yamax SW200 pedometer (Yamax Corporation, Tokyo, Japan), and ActiGraph GT3X (ActiGraph LLC,

Pensacola, FL, USA) as the gold standard step counters. A study presented by Lauritzen [22], investigated the accuracy of the commercially available trackers (eg, Fitbit) during low-intensity activities in older adults with reduced mobility. However, the study focused on a small number of participants (ie, 5 to 7 in each activity group) and wearing sites. Furthermore, although activity trackers are designed to be worn on certain positions on the body, the relationship between the wearing site and types of PAs that are best tracked by activity trackers is unknown till date. Prior research studied potential impacts of wearing site on the performance of the PA trackers [26,27]. Furthermore, the effect of sensor localization on activity monitoring performance has been studied in the past [28,29]. However, such studies did not investigate the influence of the wearing site of activity trackers on step count accuracy. By analyzing step count data gathered from trackers worn on different body positions, we can demonstrate if the wearing site can improve the step count accuracy for one or a subset of activities.

Our primary focus in this paper is to evaluate the reliability of commercially available trackers during low-intensity PAs and those activities requiring assistive tools. Specifically, our goals are to (1) determine the accuracy of 3 Fitbit devices, Zip, One, and Flex, worn at different wearing sites including pants pocket, chest, and wrist during walking at 2.5 km/h, 5 km/h, and 8 km/h performed by healthy adults on a treadmill; (2) determine the accuracy of the Fitbit trackers worn at different sites (ie, pants pocket, chest, and wrist) during real-life activities including walking with a shopping cart, walking with a walker, and eating; and (3) examine whether the intensity of PAs impacts the optimal wearing site of the tracker.

## Methods

### Pre-Study Experiment

Fitbit trackers use micro-electro-mechanical systems (MEMS) three-axis accelerometers to capture motion signals from users. It is important to mention that any disagreement among step numbers reported by the trackers was due to the wearing site of the tracker rather than the embedded signal-processing algorithm that infers step counts from the accelerometer signals. Therefore, prior to the main experiment, we performed a series of experiments to identify if the step counting algorithms of different trackers were similar in their performance while worn on the same wearing site.

The wearing site of each tracker was determined based on their specifications and convenience. Fitbit Flex is typically worn on the wrist. The extra movements of the upper body can contribute to an inability to detect steps correctly using Flex. To solve this problem, one can change the hand settings from “non-dominant” to “dominant.” The dominant option in Fitbit decreases the sensitivity of step counting [30]. We activated the dominant hand option on Fitbit Flex for left-handed participants. Fitbit Zip is often worn on locations such as a shirt pocket, bra, pants pocket, belt, and waist. Fitbit One can be worn comfortably in a pocket or on a bra.

In this experiment, one of the participants wore Fitbit Zip, One and Flex on the same wearing site for 4 hours performing daily living routines in a free-living setting. The experiment was repeated 3 times with the following scenarios: wearing all 3 Fitbit trackers (1) on the left wrist, (2) on the left pants pocket, and (3) on the chest. After each experiment, we compared the number of the steps each tracker counted in each 5-minute interval with those of the other trackers. The 5-minute interval is the shortest time interval in which the Fitbit tracker numbers can be obtained through the online dashboard [31].

### Main Experiment

We conducted a study with healthy adults who performed 6 PAs while wearing 3 different Fitbit trackers including Zip, One, and Flex on 3 different sites including chest, pants pocket, and wrist. Two sets of PAs were included in our experiment, including (1) walking on a treadmill at 3 different speeds, 2.5 km/h, 5 km/h, and 8 km/h and (2) real-life activities including walking with a shopping cart, walking with a walker, and eating an apple while sitting. In the treadmill experiment, participants were asked to walk on a treadmill at 3 different speeds for 5 minutes each. Our goal was to investigate the performance of the trackers in normal walking activities. In the shopping cart and walker experiments, participants walked with a shopping cart and a walker for 5 minutes each. In the eating activity, participants were asked to eat an apple while sitting on a chair.

**Figure 1:**
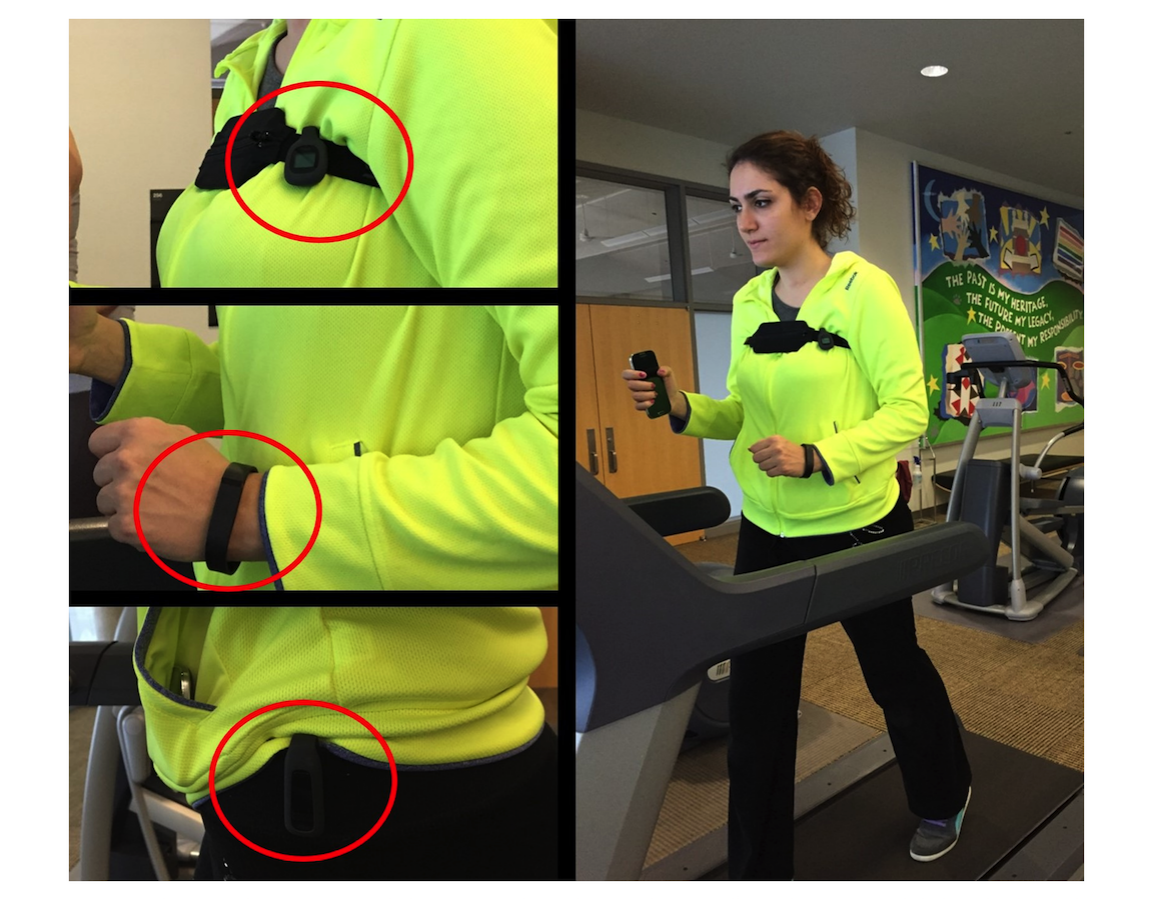
A participant wearing 3 trackers on the wrist, chest, and pants pocket while walking on a treadmill.

To capture data from participants, 2 data collection methods were used: a motion sensor based activity tracker for recording the number of steps and a camera to record videos to keep track of the actual number of steps. In our analysis, the steps measured by the trackers were compared against the gold standard number extracted from the videos. We set the dominancy option of the Fitbit Flex of the left-handed and right-handed participant to “dominant hand” and “non-dominant hand,” respectively. During the eating experiment, Fitbit Flex was worn on the dominant wrist. Therefore, we set its dominancy option to “dominant hand” for all the participants during the eating experiment. Figure 1 shows a participant wearing the trackers on left wrist, chest, and left pants pocket during a treadmill experiment.

### Recruitment and Participants

The study protocol was reviewed and approved by the Washington State University (WSU) Institutional Review Board (IRB). Inclusion criteria included the absence of gait affecting conditions such as fractures and broken bones as well as neurological impairments. Exclusion criteria included inability to walk with an assistive device, inability to walk on treadmill, and inability to conduct 30 minutes of light to moderate PA (MET<6) with multiple rests in between. Prior to data collection, all participants were informed by the study coordinator regarding the study aims, testing procedure, and methods. Participants completed an eligibility questionnaire regarding physical condition, age, and gender. The participants were recruited through direct contact as well as through an advertisement at the WSU School of EECS (Electrical Engineering & Computer Science).

### Statistical Analysis

We first assessed systematic differences between trackers on 3 wearing sites and the gold standards by a one-way analysis of variance (ANOVA) test. We also defined 2 error parameters: *errors per minute* and *error rate*. Error per minute is defined as the difference between the number of steps recorded by the trackers and the actual number of steps in 1 minute. Error rate refers to the percentage of the misestimated. We note that for the eating experiment, given that the actual number of steps is 0, the absolute error is not reported.

Second, we evaluated the test-retest reliability of the tracker on each wearing site by computing the intraclass correlation coefficient (ICC) (2-way random, absolute agreement, single measures with 95% CI). We used common cut-off points for reliability assessment. The cut-off points were >0.90 (excellent), 0.75-0.90 (good), 0.60-0.75 (moderate), and <0.60 (low) [5].

## Results

### Pre-Study Results

A total of 15 healthy adults, including 7 females and 8 males, aged between years 21 and 31, were recruited to participate in this experimental study. Table 1 shows demographic information as well as PA statistics for the participant groups.

Table 2 shows the results of the *t* test analysis on pairs of the Fitbit trackers and gold standard during the pre-study test. The results show no significant difference in the number of steps recorded during the test experiment. Therefore, we conclude that any possible differences in the number of steps reported by various trackers used in the main study must be due to the tracker’s wearing site for that specific task.

### Main Results

#### Descriptive Statistics Analysis

**Figure 2:**
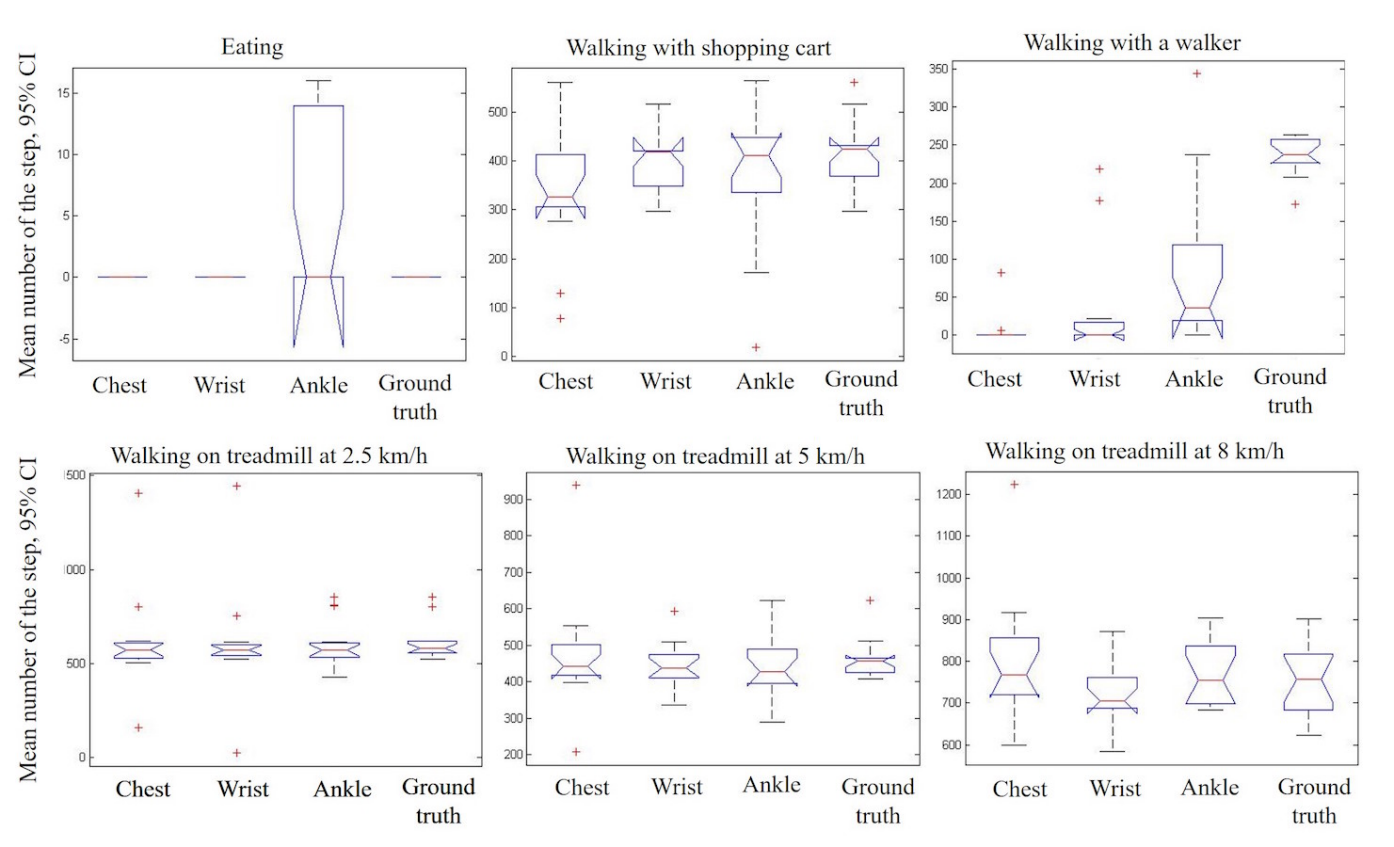
The result of the ANOVA tests on the steps recorded by trackers worn on different wearing sites (chest, pocket, and wrist) with the gold standard number of steps.

Figure 2 shows the results of the ANOVA test (95% CI 0.55-1.00) on the mean value of the step counts captured by the trackers on each wearing site compared to gold standard values. There are no significant differences in recorded steps in each of the 3 wearing sites (chest, pants pocket, and wrist) for walking on treadmill and shopping cart experiments. In the walker experiment, all trackers exhibit a significant difference from the actual step count (*P*<.001). In the eating experiment, the wrist tracker occasionally counted steps due to the hand movements while eating the apple.

#### Systematic Difference and Mean Absolute Error Analysis

**Figure 3:**
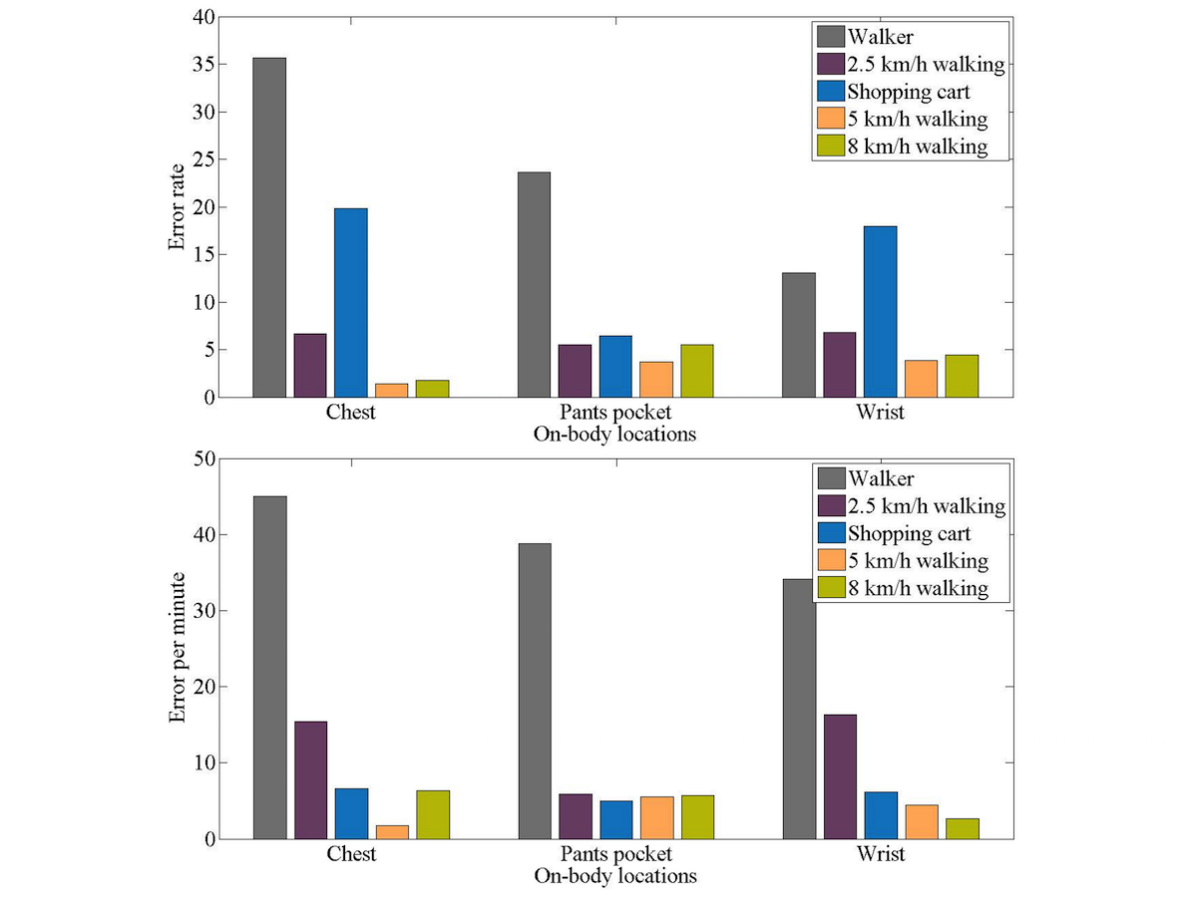
Error rate (left) and error per minute (right) of activity trackers in different wearing sites during the activities of daily living except eating.

The performance of the trackers in step counting was compared using the average error rate and error per minute values of all the participants during the experiment. Figure 3 illustrates the performance of the trackers in different wearing sites during activities of daily living. This figure compares error per minute and error rate in counting steps. The error rate and error per minute metrics for the various trackers are detailed in Table 3.

Furthermore, the chest tracker recorded 0 steps during the eating experiment, the wrist tracker counted 4.8 steps per minute in the eating experiment, and the pants pocket tracker counted 0 steps during the eating experiment.

**Table 1:** Physical and demographical characteristics of the participants.

Variables	All (N=15)	Female (n=7)	Male (n=8)
Age in years	21-31	24-26	23-31
Height (cm)	155-189	155-178	175-189
Body mass (kg)	47-86	47-75	65-86
Body Mass Index (kgm^−2^)	19.0-24.8	19.0-24.8	20.1-24.8
Average number of steps taken in treadmill experiments	610	583	643
Average number of steps taken in walker experiment	236	231	240
Average number of steps taken in shopping cart experiment	417	400	418

**Table 2:** *t* test results on the number of the steps different Fitbit trackers count when used on the same wearing sites.

Wearing site	Trackers	Hypothesis	Probability
Wrist	Zip and One	0	.65
	Zip and Flex	0	.79
	One and Flex	0	.83
Chest	Zip and One	0	.69
	Zip and Flex	0	.83
	One and Flex	0	.85
Pants pocket	Zip and One	0	.84
	Zip and Flex	0	.78
	One and Flex	0	.93

**Table 3:** Error rate and error per minute values for trackers.

Wearing site	Activity	Error rate	Error per minute
Wrist	Walking with walker	73.1%	34.2
	Walking at 2.5 km/h	6.8%	6.2
	Walking with shopping cart	19.8%	16.4
	Walking at 5 km/h	3.9%	4.4
	Brisk walking at 8 km/h	4.4%	2.6
Chest	Walking with walker	95.6%	45.0
	Walking at 2.5 km/h	6.7%	6.6
	Walking with shopping cart	19.8%	15.4
	Walking at 5 km/h	1.4%	1.8
	Brisk walking at 8 km/h	1.8%	6.3
Pants pocket	Walking with walker	83.6%	38.8
	Walking at 2.5 km/h	5.5%	5.0
	Walking with shopping cart	6.4%	5.9
	Walking at 5 km/h	3.7%	5.5
	Brisk walking at 8 km/h	5.5%	5.7

**Table 4:** Intraclass correlation coefficients between the wearing sites and gold standard values.

Wearing site	Activity	ICC	95% CI
Wrist	Treadmill walking at 2.5 km/h	0.76	0.61-0.94
	Treadmill walking at 5 km/h	1.07	0.91-0.99
	Treadmill brisk walking at 8 km/h	1.07	0.90-0.98
	Walking with the walker	<0.01 (0.003)	0.68-0.99
	Walking with the shopping cart	0.10	0.73-0.87
	Eating	0.00	0.67-1.00
Chest	Treadmill walking at 2.5 km/h	0.70	0.56-0.93
	Treadmill walking at 5 km/h	1.37	0.97-0.99
	Treadmill brisk walking at 8 km/h	0.68	0.55-0.92
	Walking with the walker	<0.01 (0.006)	0.87-0.97
	Walking with the shopping cart	0.61	0.74-0.79
	Eating	N/A^a^	N/A
Pants pocket	Treadmill walking at 2.5 km/h	0.91	0.73-0.97
	Treadmill walking at 5 km/h	0.83	0.67-0.95
	Treadmill brisk walking at 8 km/h	0.71	0.58-0.93
	Walking with the walker	0.02	0.82-0.95
	Walking with the shopping cart	0.74	0.83-0.87
	Eating	N/A	N/A

^a^N/A: not available.

#### Correlation Analysis

Table 4 shows the ICC between the trackers on each wearing site and the gold standard for each PA type. The ICC values ranged from 0.56 (chest) to 0.97 (pants pocket) for walking on the treadmill at 2.5 km/h, 0.55 (chest) to 0.99 (wrist) for walking on the treadmill at 5 km/h, 0.58 (pants pocket) to 0.98 (wrist) walking on the treadmill at 8 km/h, 0.68 (wrist) to 0.99 (wrist) for walking with the shopping cart, 0.74 (chest) to 0.87 (wrist) for walking with the walker, and 0.67 (wrist) to 1.00 (wrist) for eating.

## Discussion

We evaluated the performance of 3 Fitbit trackers during 2 sets of PAs: (1) Easy to monitor activities such as walking on a treadmill, and (2) real-life activities that are potentially harder to monitor such as walking with a walker, walking with a shopping cart, and eating. Among these activities, the ones performed with lower intensity such as walking with a walker and walking at 2.5 km/h may represent some of the activities that older adults perform regularly. While we acknowledge that a limitation of our study is the lack of older adult participants, this study may have implications for utilizing activity trackers in populations that routinely perform PAs with a lower intensity or those that involve carrying walking aids.

The fact that the utilized trackers in this study were most accurate during treadmill walking can be explained by the controlled nature of human gait during treadmill walking as well as the fact that in normal gait the body will not exert extra movements to control the balance. As a result, trackers can easily detect each strike during treadmill walking. We also observed that decreasing or increasing the walking pace from normal speed reduces the accuracy of step counting. In non-treadmill activities, step detection becomes less accurate because movement patterns deviate from typical human gait patterns.

Our systematic differences analysis revealed that the intensity of PA impacts the choice of optimal wearing site of the tracker. Our results showed that the chest was the best site for more intense activities such as moderate walking at 5 km/h and brisk walking at 8 km/h, while the same site was least accurate in low intensity activities such as walking at 2.5 km/h and walking with the walker. This is consistent with the results by other researchers who reported the waist as the least accurate site for tracking low-intensity activities [4]. Furthermore, our results show that wrist was the most promising site during less intense activities such as walking at 2.5 km/h and walking with a walker. These results confirm prior findings by Diaz et al [15], who discovered that a wrist tracker had the biggest difference during slow, moderate and brisk walking on the treadmill. Moreover, our results showed that the pants pocket was a better wearing site in terms of step counting accuracy while pushing a shopping cart. Yet, this wearing site was least accurate during intense activities. This finding is also consistent with the results obtained in several prior studies such as a study by Mammen et al [4].

Looking at the result of the error-per-minute for all of the activities, one could conclude that there are 2 triggers to accurately detect the steps in PA. First, more intense activities are easier to detect. Second, steps in activities that better imitate normal walking such as walking at 5 km/h and 8 km/h are better identifiable compared to activities with abnormal walking patterns such as walking with a walker. Crouter et al [16], examined the accuracy of 5 electronic pedometers and found that pedometers are less accurate at slower walking speeds. In another work performed by Thorup et al [23], Fitbit Zip demonstrated high accuracy (absolute error <3%) on the walking speeds of 3.6 km/h and higher.

In our ICC analysis, the wrist site (ie, Flex) showed good-to-excellent correlation (ICC>0.75) during treadmill walking. Several prior studies indicated good correlation (ICC 0.75-0.90) between the wrist tracker (Flex) and the gold standard as well as the experiments conducted by Kooiman et al [5] on the accuracy of 10 consumer level activity trackers.

A study by Beets et al [32] on the accuracy of the pedometers in youth with developmental disabilities indicated a low correlation (ICC<0.60) during a shopping cart experiment [7,8]. In our study, the site pants pocket (ie, One) demonstrated good-to-excellent correlation (ICC>0.75) as well. This amount of correlation is smaller than the prior results by Beets et al. They identified that a hip worn tracker demonstrated excellent correlation (ICC 0.97-0.99) with the gold standard values [8]. This can be explained by the fact that a hip tracker is potentially more stationary in body coordinates compared to a pants pocket tracker than can be potentially loose during human gait. We observed a moderate correlation (0.60<ICC<0.75) with the gold standard while walking with the shopping cart.

In our analysis, the chest site had a moderate correlation in treadmill walking at 2.5 km/h and 5 km/h, while it was excellent during walking on the treadmill at 8 km/h. In a research study, the upper body Fitbit tracker had a low correlation (ICC 0.1-0.4) for low-intensity treadmill walking (1.7-2.7 km/h) [4]. It showed moderate correlation (0.60<ICC<0.75) with gold standard during the shopping cart experiment.

Our data show that accelerometer-based trackers provide reliable measures for moderate and brisk walking on treadmill. The performance of these trackers decline as the walking speed increases or decreases from the normal walking pace. Moreover, the accelerometer-based trackers demonstrate a major performance drop in step detection during activities that deviate from a normal walking pattern. An example of such abnormal walking patterns is when walking with a walker. This may suggest that utilizing commercially available trackers in studies that involve low-speed activities and those involving walking aids requires either careful selection of an activity tracker robust to such activities or exclusion of participants whose routine behavior involves these activities.

Furthermore, our result, in particular those related to walker and shopping cart experiments, may suggest a need for developing more advanced algorithmic solutions that consider tracker wearing sites as well as activity intensity into account while computing step counts. Accelerometer-based step detection algorithms usually try to find one stride by detecting the minimum and maximum peaks from the accelerometer sensor signal. Finding a stride pattern in the acceleration signal of irregular walking patterns such as walking with a walker or a shopping cart can be more challenging that requires real-time adaptation of the underlying algorithms to personalize the step counting methods for each individual and with respect to the changing context of the user [23]. Developing such an algorithm first requires detecting the user’s activity type in real-time and second identifying the wearing site of the sensor to obtain the highest accuracy. Using advances discussed in recent studies, we are able to locate the sensor on the user’s body coordinate by identifying the wearing site [28,29]. Furthermore, the study presented by Krishnan et al [33]and other researchers on activity recognition suggest that we can use machine learning algorithms to identify PA types and wearing sites of the sensors from accelerometer sensor data.

## Abbreviations

ICC: intraclass correlation coefficient
MEMS: micro-electro-mechanical systems
PA: physical activity
